# Emphysematous Osteomyelitis of the Lumbar Spine and Sacrum After Liver Transplantation: A Case Report

**DOI:** 10.7759/cureus.63748

**Published:** 2024-07-03

**Authors:** Rio Kou, Yasuyuki Onishi, Satoshi Ogiso, Shinichi Kai, Yuji Nakamoto

**Affiliations:** 1 Diagnostic Imaging and Nuclear Medicine, Kyoto University, Kyoto, JPN; 2 Surgery, Kyoto University, Kyoto, JPN; 3 Anesthesia, Kyoto University, Kyoto, JPN

**Keywords:** septic shock, pumice stone sign, liver transplantation, immunosuppression, emphysematous osteomyelitis

## Abstract

Emphysematous osteomyelitis (EO) is a rare and potentially fatal disease that often occurs in patients with underlying conditions, most commonly diabetes mellitus. Herein, we report a case of a 62-year-old man who presented with fever, tachycardia, and hypotension 112 days after liver transplantation. Blood tests revealed an increased inflammatory response. Computed tomography demonstrated clusters of small gas collections in the first and second lumbar vertebral bodies and the right sacral ala, a finding characteristic of the pumice stone sign of EO. Septic shock due to EO was diagnosed. The patient responded well to treatment and recovered from the infection. This case suggests that the immunosuppressive state after liver transplantation is a risk factor for EO.

## Introduction

Emphysematous osteomyelitis (EO) was first reported in 1981 and is characterized by intraosseous gas produced by infection [1]. It has been reported in patients ranging from 13 to 91 years of age, with no gender differences [2-4]. In most cases, EO occurs in patients with underlying diseases such as diabetes mellitus and malignancy [2]. The pelvis, vertebrae, and femur are commonly affected [5]. *Escherichia coli* and *Klebsiella pneumoniae* are common causative organisms of EO [6]. Hematogenous spread is the main route of infection [2]. EO is a life-threatening disease with a mortality rate of 34.8% and requires antimicrobial therapy and surgical treatment [7]. Herein, we report a case of EO that developed after liver transplantation.

## Case presentation

A 62-year-old man underwent living donor liver transplantation for alcoholic cirrhosis. Postoperatively, he was administered an intravenous infusion of the immunosuppressant tacrolimus with a target concentration of 15-18 ng/mL and the antifungal drug micafungin at 100 mg/d. He was also administered the immunosuppressant mycophenolate mofetil via enterostomy at 1 g/d. The patient experienced rejection of the transplanted liver, which improved with steroid pulse therapy. Methylprednisolone was administered intravenously from postoperative days (POD) 17 to 32, tapering from 665 mg/d to 20 mg/d. From POD 33, intravenous methylprednisolone was replaced with oral prednisolone and tapered gradually from 20 mg/d. The patient also developed pneumonia and cholangitis, which improved with intravenous antibacterial and antifungal treatments. On POD 32, tacrolimus was changed to oral administration with a target trough concentration of 10-12 ng/mL. On POD 35, mycophenolate mofetil was changed to 1 g/d orally. On POD 39, micafungin was changed to 100 mg/d orally. On POD 52, the patient was transferred to another hospital for rehabilitation, which proceeded well, while tacrolimus blood levels and liver enzymes were monitored.

**Figure 1:**
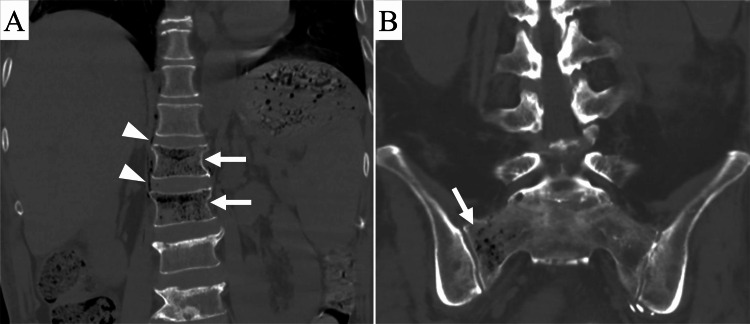
Computed tomography (CT) images of the lumbosacral spine obtained 112 days after liver transplantation. (A) Coronal CT image of the lumbar spine shows clusters of small intraosseous gas (arrows) in the first and second lumbar vertebral bodies, extraosseous gas (arrowheads) around the vertebral bodies, and compression fractures of the first and second lumbar vertebral bodies. (B) Coronal CT image of the sacrum shows clusters of small intraosseous gas (arrow) in the right sacral ala.

On the morning of POD 112, he developed a fever > 38°C, hypotension, and tachycardia with a heart rate > 130 bpm. He also complained of back pain. Blood tests showed an increased inflammatory response (C-reactive protein: 14 mg/dL; white blood cell count: 12180/µL) and decreased renal function (creatinine: 3.09 mg/dL). Computed tomography (CT) revealed clusters of small gas collections in the first and second lumbar vertebral bodies and the right sacral ala, exhibiting the pumice stone sign characteristic of EO (Figure 1).

Gas was also observed around the vertebral bodies, and compression fractures were seen in the first and second lumbar vertebral bodies, likely resulting from EO. Cortical destruction was not observed. A diagnosis of septic shock due to EO was made, and the patient was urgently transferred to the intensive care unit at our hospital. Upon arrival, he experienced cardiopulmonary arrest, for which cardiopulmonary resuscitation, including chest compression and artificial respiration, was performed. Blood transfusions, fluid transfusions, and intravenous infusions of a vasopressor, meropenem 1 g/d, and vancomycin 1 g/d were started. His systolic blood pressure subsequently recovered to 120 mmHg. The patient’s cardiopulmonary arrest was thought to result from tachycardic atrial fibrillation triggered by septic shock. To treat sepsis, oral prednisolone was replaced with intravenous cortisol, fluconazole was replaced with intravenous micafungin 100 mg/d, and other oral medications were temporarily withdrawn. Due to decreased renal function and severe acidosis, continuous hemofiltration was initiated. Thereafter, his systolic blood pressure remained at 140 mmHg. Based on the results of blood sampling on POD 114, the patient was considered to be recovering from sepsis, leading to the withdrawal of continuous hemofiltration.

**Figure 2:**
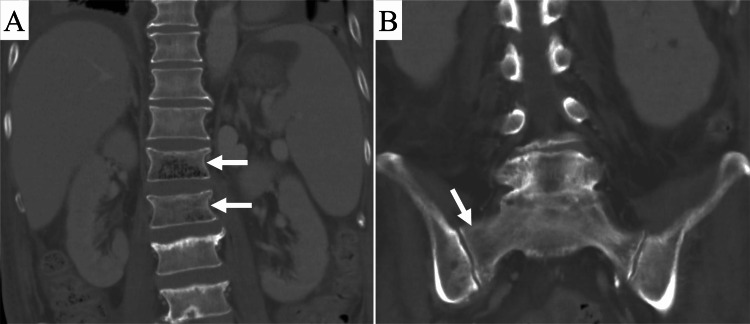
Computed tomography (CT) images of the lumbosacral spine obtained 118 days after liver transplantation. (A) Coronal CT image of the lumbar spine shows a decrease in intraosseous gas (arrows) in the first and second lumbar vertebral bodies. No extraosseous gas is observed. (B) Coronal CT image of the sacrum shows no intraosseous gas in the right sacral ala (arrow).

Subsequently, blood and urine cultures on the day of emergency admission grew *Klebsiella pneumoniae*. EO was thought to have occurred via hematogenous spread from a urinary tract infection. Therefore, micafungin and vancomycin were stopped, and administration of meropenem 4 g/d was started. The patient was discharged from the intensive care unit on POD 118 after his general condition improved, and immunosuppressive drugs were resumed. A CT scan on the same day showed a reduction in intraosseous and extraosseous gas (Figure 2).

The patient was transferred back to the referral hospital at POD 138 after confirming that his clinical symptoms had stabilized, and he was treated conservatively with a back brace for vertebral compression fractures.

## Discussion

Many patients with EO have significant underlying medical conditions. Conditions like diabetes mellitus, malignancy, alcohol abuse, and steroid use are known to predispose individuals to EO, with diabetes mellitus being the most common [2,6,7]. In the present case, EO occurred while the patient was taking immunosuppressive drugs after liver transplantation, suggesting that immunosuppression was the most likely cause. The period between one and six months after organ transplantation poses the risk of developing opportunistic infections [8]. *Klebsiella*, identified as the causative organism in this patient, is also known to cause opportunistic infections in immunocompromised patients [9]. To our knowledge, there have been no previous reports on EO after liver transplantation, making our case noteworthy.

EO is characterized by aggressive gas-forming infections that rapidly destroy the intramedullary bone marrow and form numerous air-dense foci of varying size and shape [5]. CT imaging shows clusters of multiple, irregular intraosseous gas pockets < 1 cm in size, referred to as the pumice stone sign [5]. In this case, vertebral compression fractures were seen, a complication reported in several studies of EO [6,10,11]. The infection weakens the bone, leading to fragility and subsequent compression fractures.

Vertebral compression fractures commonly present as linear or semi-lunar radiolucent shadows on plain radiographs, known as the intravertebral vacuum cleft sign [12]. Although both the intravertebral vacuum cleft sign and the pumice stone sign involve intraosseous gas, they are different in terms of gas distribution. Most vertebral compression fractures without infection can be managed conservatively, whereas EO requires aggressive treatment, such as antimicrobials and surgical debridement. Therefore, distinguishing these two conditions is crucial for appropriate management.

The pelvis (38%), vertebrae (32%), and femur (24%) are commonly reported sites of EO [5]. In the present case, lesions were found at two of these three sites. Numerous studies have documented EO cases involving multiple locations, such as the vertebral body and pelvis, or the pelvis and femur [5,6]. This contrasts with the general understanding that osteomyelitis rarely affects more than one bone [13]. Clinicians should conduct thorough physical examinations and imaging assessments, recognizing that EO can occasionally occur at multiple sites.

## Conclusions

We reported a case of EO occurring in an immunosuppressed state following liver transplantation. It is important to understand that the pumice stone sign is the distinctive feature of EO and that bone lesions can occasionally occur at multiple sites. This case suggests that an immunosuppressive state after liver transplantation may be a risk factor for the development of EO.
